# A heuristic model for collaborative practice—part 2: development of the collaborative, dialogue-based clinical practice model for community mental health and substance abuse care

**DOI:** 10.1186/s13033-020-00377-4

**Published:** 2020-06-09

**Authors:** Rolf Sundet, Hesook Suzie Kim, Bengt Eirik Karlsson, Marit Borg, Knut Tore Sælør, Ottar Ness

**Affiliations:** Faculty of Health and Social Sciences, Department of Health, Social & Welfare Studies, University of South-Eastern Norway, PO Box 235, 3603 Kongsberg, Norway

**Keywords:** Collaborative practice, Mental health and substance abuse, Community mental health and substance abuse practice, Person/professional collaboration, Dialogue-based, Clinical practice model for mental health and substance abuse care

## Abstract

**Background:**

Various models for collaborative practice in mental health care incorporating the perspectives of service-user participation and collaboration in the care have been developed. However, the emphasis in these practice models has not been on identifying specific features of “how” collaboration and service-user participation can occur and be nurtured. This suggests a need for a collaborative practice model that specifies essential strategies operationalizing the tenets of service-user participation and collaboration applicable in mental health and substance abuse (MHSA) care.

**Methods:**

A double helix approach of coalescing theoretical ideas and empirical findings to develop a practice model that is applicable in MHSA practice. A theoretical analysis is carried out to identify the critical, foundational elements for collaborative practice in MHSA practice, and has identified the philosophical-theoretical orientations of Habermas’ theory of communicative action, Bakhtin’s dialogicality, and the philosophy of personhood as the foundational features of collaboration. This base is juxtaposed with the results of a qualitative meta-analysis of 18 empirical articles on collaboration in MHSA to advance a collaborative practice model specifically in the domain of service user/professional collaboration.

**Results:**

“The collaborative, dialogue-based clinical practice model” (CDCP Model) for community mental health care is proposed, within the structure of four main components. The first specifies the framework for practice that includes person-centered care, recovery-orientation, and a pluralistic orientation and the second identifies the domains of collaboration as service user/professional collaboration, inter-professional collaboration, and service sector collaboration. The third identifies self-understanding, mutual understanding, and shared decision-making as the essential principles of collaboration. The fourth specifies interactive-dialogic processes, negotiated-participatory engagement processes, and negotiated-supportive processes as the essential strategies of collaboration applicable in service user/professional collaboration which were extracted in the empirical work. An illustration of the CDCP Model in a clinical case is given.

**Conclusions:**

The CDCP Model presented fills the gap that exists in the field of community MHSA practice regarding how to operationalize systematically the tenets of person-centeredness, recovery-oriented, and pluralism-oriented practice in terms of user/professional collaboration.

## Introduction

Recovery-oriented practices have become the desired modes of mental health and substance abuse (MHSA) practice currently, and there have been various national and international efforts to implement and integrate this practice perspectives into MHSA services [1–9]. The major tenet of recovery-orientation is citizenship and human rights, social inclusion and empowering partnerships between the person and the professionals in terms of setting personal goals and making choices and decisions [6, 10–13]. Collaborative relationships and therapeutic alliances in mental health care have been found to have a positive impact on the person-centered and recovery-oriented processes of care and service user outcomes [14, 15]. Various models for these modes of practice in mental health care have been developed and implemented incorporating the perspective of service-user participation and collaboration in the care, for example, Implementing Recovery through Organizational Change [16, 17], Care Programme Approach/Care and Treatment Planning of England and Wales [9], the Recovery-oriented Behavioral Health Care [18], and many others developed in various countries [19]. However, the emphasis in these practice models has not been on identifying specific features of “how” collaboration and service-user participation need to occur and be nurtured, especially in terms of dialogical processes applicable in the partnerships and collaboration. This suggests a need for a collaborative practice model that is an overlay on these two modes of MHSA care so that the tenets of service-user participation and collaboration can be actualized in practice.

Ness et al. [20] proposed a framework for collaborative practice for community mental health care that identifies and describes the key orientations, components, principles, and processes for collaborative practice. This paper in a series of our work in advancing collaborative practice in community mental health care elaborates, expands, and refines this framework with a specific focus on formalizing a person/professional collaborative practice by articulating specific collaborative strategies. The aim is to present a clinical collaborative practice model for application in practice by professional providers in mental health and substance abuse (MHSA) practice. This proposed model anchored in the framework proposed by Ness et al. [20] extends it and elaborates specific collaborative, dialogical strategies applicable in the collaborative processes for and with MHSA service users drawing upon the results of a meta-synthesis of our empirical work presented in Part One of the papers in the series. The objective of this paper is a specification and elaboration of “the collaborative, dialogue-based clinical practice model” (CDCP Model) as the next step of specifying the collaborative practice framework presented by Ness et al. [20], which can be applied as the representative model of MHSA practice incorporating the tenets of person-centered care, recovery-orientation, and pluralistic-orientation.

A precursory clarification regarding the use of specific terms in this paper is in order. Among the terms such as client, patient, service user, and consumer we use the term “person” or “user” to refer to the citizen in need of healthcare service for MHSA care, while among the terms such as clinician, professional, therapist, or service provider, we use the term “professional” to refer to the person providing clinical, professional health care services directly to users. In addition, we use the term “clinical practice” to encompass the work of healthcare that involves therapy, care, and services for people in need of health care.

## Background and foundations

The person/professional collaboration is one sector of the comprehensive features of collaborative healthcare practice, which is centrally couched within the clinical practice processes to bring about improved client outcomes in general healthcare practice but more specifically in MHSA practice. As the clinical practice processes of MHSA care is critically embedded in interactive-communicative processes between users (and often with users’ family members or significant others) and professionals, collaboration has been identified as one of the key aspects of theses interactive processes. Various collaborative and user-involvement practice models in mental health care suggest positive user outcomes such as self-management, personalized care, and better functioning [21–23]. The core of the clinical process in community MHSA practice both in terms of singular clinical encounters and continuing clinical relationships is configured by the goal of addressing the user’s MHSA problems successfully with recovery-orientation through the process that is interactive and relationship-based, upholding the values of human dignity, autonomy, and singularity. The key principles of collaboration thus include (a) shared value in collaboration, (b) equalization of power in relationships, (c) mutual trust and respect, (d) sharing of visions and goals, (e) developing and maintaining interpersonal alliance, (f) self and mutual understanding, and (f) open communication [24, 25]. We utilize three strands of philosophical-theoretical orientations to make up the foundational features of collaborative practice in MHSA practice, including Habermas’ theory of communicative action, Bakhtin’s dialogicality, and the philosophy of personhood specifying the concepts of dignity, autonomy, and singularity.

### Habermas’ theory of communicative action

In the theory of communicative action, Habermas [26] proposes “communicative action” oriented to understanding and consensus as the basis for language-oriented forms of social life, differentiating it from “strategic action” oriented to success for a speaker’s goals. He focuses on the features of speech acts between people as the basis for arriving at mutual understandings through consensus on validity claims of truth, rightness, and truthfulness. Communicative action to Habermas [27] is a form of social interaction in which the participants engage together toward reaching consensus through coordinating their validity claims. Social interaction between people thus consists of both strategic action and communicative action, and it is through communicative action people can arrive at mutual understanding. Habermas ([27], p. 58) states:

Whereas in strategic action one actor seeks to influence the behavior of another by means of the threat of sanctions or the prospect of gratification in order to cause the interaction to continue as the first actor desires, in communicative action one actor seeks rationally to motivate another by relying on the illocutionary binding/bonding effect (Bindungseffekt) of the offer contained in his speech act.

Mutual understanding, therefore, is achievable only through communicative action among participants, which is intentionally oriented to moving toward coordination and consensus. It is the first step toward working collaboratively by attaining understandings about each other’s goals, intentions, and meanings. The mutual understanding between users and professionals is specifically critical as there can be differences and disparities in understanding users’ problems, clinical approaches, and modes of arriving at personal and clinical goals. Mutual understanding is the foundation for people to acknowledge and work with differing perspectives, values, motivations, and expertise. Mutual understanding in user/professional relationships is the base for building mutual respect for the knowledge and experiences of professionals and users as critical for the work of users’ recovery. The pre-condition for communicative action is what Habermas calls “ideal speech situation” in which participants have speech competence and are free to express their opinions and ask questions without constraints from repression, coercion, or inequality [26]. This means that constraint-free communication can only occur when participants are free to express their ideas, feelings or judgments without being internally (that is, by one’s self) or externally coerced. Although Habermas’ theory of communicative action is fundamentally oriented to social integration at the societal level, it is applicable in the concept of collaboration focusing on its foundation on “talk” (i.e. communicative action).

### The dialogical perspective of Bakhtin in MHS

The dialogical perspective in Bakhtin`s ([28], p. 293) work is:

The single adequate form for verbally expressing authentic life is the open-ended dialogue. Life by its very nature is dialogic. To live means to participate in dialogue: to ask questions, to heed, to respond, to agree, and so forth. In this dialogue a person participates wholly and throughout his whole life: with his eyes, lips, hands, soul, spirit, with his whole body and deeds. He invests his entire self in discourse, and this discoursed enters into the dialogical fabric of human life, into the world symposium.

In MHSA this dialogical perspective is especially championed by Finish psychologist Seikkula and collogues [29, 30] through the development of Open Dialogues (OD) as a manner of meeting and working with persons in a psychotic crisis. In OD the alternating roles of speaker and listener are based in an understanding that the minimum condition for dialogue is a process of mutual turn-taking [25] between these two positions. The dialogical perspective or dialogicality is about the face-to-face interplay between interlocutors and the utterances that take place in their turn-taking [31]. In dialogical encounters, the concepts of polyphony [28] and voice [32] together take a special position. Seikkula and Arnkil write: “The social reality is polyphonic: it speaks in many voices. In every social situation, a variety of different voices are present. The term “voice” refers both to the speaking subject and to the consciousness” ([29], p. 99), and as such “…it is concerned with the broader issue of a speaking subject’s perspective, conceptual horizon, intention, and world view” ([31], p. 51). An utterance is always started from a location addressed at another person and so there is always more than one voice involved. Dialogicality then is multi-voiced [31]. This multi-voiceness is expressed through the concept of polyphony. Analyzing the work of Dostoevsky, Bakhtin ([28], p. 6) writes:

*A plurality of independent and unmerged voices and consciousnesses, a genuine polyphony of fully valid voices is in fact the chief characteristic of Dostoevsky`s novels.* What unfolds in his work is not a multitude of characters and fates in a single objective world, illuminated by a single authorial consciousness; *rather a plurality of consciousnesses, with equal rights and each with its own world*, combine but are not merged into a unity of the event.

In this perspective, the polyphonic implies that outer and inner dialogues, from different positions of all communicating partners, exist and are active in any dialogical moment. The dialogical event and moment are characterized by difference. Bakhtin ([33], p. 88) writes:

…what is important from the standpoint of the productiveness of the event of my life is not the fact that, besides myself, there is one more person of essentially the same kind (two persons), but the fact that the other is for me a different person. And in this sense his ordinary sympathizing with my life is not a merging of the two of us into a single being and is not a numerical duplication of my life, but constitutes an essential enrichment of the event of my life, because my life is co-experienced by him in a new form, in a new axiological category—as the life of another, different human being.

This brings us to the question of how to relate, see, and understand what it means to be another person? How to relate to the other? What is Otherness? Emmanuel Levinas connects Otherness to a type of alterity that cannot be conceptualized. Alterity that “eludes the philosophical thought evolving under the hegemony of ´the same ([34], p. 2). Levinas states that “The primacy of the same was Socrates teaching: to receive nothing of the Other but what is in me” ([35], p. 43).

Opposition in a philosophical starting point is found here, between sameness and difference. The dialogical perspective states the difference-position “…as having unconditional respect for the uniqueness of the other. Being heard, the key experience in dialogical relationships, calls for acknowledging, accepting and respecting that the other is always more than one can grasp; and responding to the unique other in the present moment” ([36], p. 141).

### The philosophy of personhood

The third theoretical foundation for collaboration is couched in the philosophy of personhood. The philosophy of personhood is oriented to upholding the essential features of being humans in terms of dignity, autonomy, and singularity of humans. The concepts of human dignity and personal autonomy founded upon Kantian philosophy are the two key fundamental values for humanity. Human dignity refers to the value of worthiness of humans with rights and respect and is the basis for the empowerment of individuals for maintaining selfhoods.

Autonomy, additionally, refers to the human capacity and right for a person to be oneself without being forcibly manipulated by others in making decisions, and to live one’s life according to one’s reasons and motives not controlled or distorted by external forces [37]. The values of human dignity and autonomy together are the basis for the relational ethics that govern social life in general as well as client/professional relationships in particular. Autonomy is the subject of debate often in the context of health care, especially in MHSA care, in which “paternalistic” approaches of professionals that can offend users’ autonomy are justified by the notion that the user is not able to decide for oneself how best to pursue one’s good. However, the governing principle for the value of autonomy goes hand-in-hand with the value of dignity as the fundamental aspects of personhood to be respected and upheld. Furthermore, autonomy can be viewed beyond that of the fundamental property of an individual by considering it relational that emphasizes the role of social relationships both in forming self-concepts relative to self-determination and in exercising self-government for the deliberation of autonomy [38]. Relational autonomy can thus be the basis for collaboration and social support in user/professional relationships.

The concept of singularity for personhood refers to the uniqueness of individuals as well as of human experiences. The concept of singularity in these two senses is paradoxically intertwined, as in Derrida’s concept of *différance* [39], with the fundamental sameness in humanity regarding the uniqueness of individuals and with the repeatability of human experiences regarding the uniqueness of events and human experiences. The singularity of personhood, therefore, embraces the uniqueness of the present that has the continuity and repeatability that are based on the fundamental nature of humanity and in human history. This philosophical stance regarding humans as a complex of valued commitments for dignity, autonomy, and singularity orients users and professionals engaged in clinical encounters to uphold the mutual humanity in their relationships.

Furthermore, personhood expressed by dignity, autonomy, and singularity configures into the sociality of individuals’ experiences as persons. Personhood therefore thus is the basis for a person to be a citizen in a society, both fulfilling the obligations of citizenship and enjoying the rights and privileges of citizenship in social life. Ponce and Rowe state that “citizenship includes basic recognition of the humanity, equal worth, and dignity of all persons and groups” ([40], p. 24). This connection between the concept of personhood and citizenship is critical in mental health to expand the realm of MHSA practice to encompass citizenship issues as well as clinical and recovery orientations.

### Summary

These three foundational positions can be summarized as (a) the communicative theory of action underlines negotiations, consensus, and the reaching of agreements through communicative actions; (b) dialogicality as the fundamental feature of human life underlines diversity, difference, and the polyphonic; and (c) the philosophy of personhood establishes autonomy, dignity, and singularity as the core values and positions that undergird what is to be a person and live a fullest possible life. Within these three sets of concepts, there is a clear tension. A tension between agreement, and as such, the value of sameness on one side and difference and diversity on the other side expressed through a concept of singularity that consolidates a radical “otherness” as the core of individuality and personhood. This echoes the work of Hannah Arendt. She states: “Plurality is the condition of human action because we are all the same, that is, human, in such a way that nobody is ever the same as anyone else who ever lived, lives or will live” ([41], p. 8). Dutch educational theorist Gert Biesta [42, 43] uses this to make a distinction between uniqueness-as-difference and uniqueness-as-irreplaceability. In the first, we all can be described through different concepts, theories, and descriptive systems, but also limited within these conceptual manners of describing and understanding the Other. Uniqueness-as-irreplaceability connects to Arendt`s plurality and the type of “otherness” that we meet in both Bakhtin and Levinas` work: That which is never met before and as such outside our descriptive and knowledge repertoire. This part brings in a fundamental uncertainty in any communicative and dialogical encounter. Relying on the sameness between what I know of similarities between persons and what I meet in the other, cannot save me from the fact that the uniqueness-as-irreplaceability always opens up for the not-met-before, the not-known and the radically new as that which will mark my encounter with the other, and therefore I can never know (anything) for certain.

The field of tensions described by bringing together these three foundations can be found in the empirical findings brought out in Part One of this series of papers from our projects. This field of tension can be seen as an ethical field because it is never given how to respond to the other in the singular meeting. This must be found out, negotiated and brought forth in the actual collaborative process.

## The Collaborative, Dialogue-based Clinical Practice (CDCP) Model

**Fig. 1:**
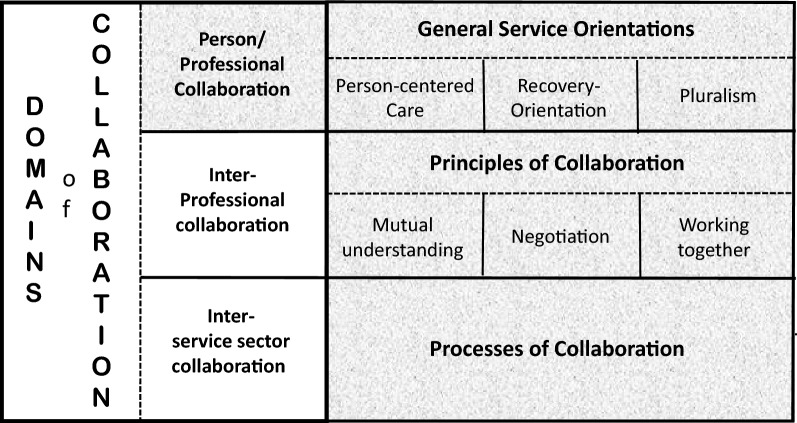
Depiction of the framework of collaborative healthcare practice

This proposed CDCP Model for community mental health care, draws from the framework of collaborative practice proposed by Ness et al. [20]. This framework is configured by four components: (a) the frame orientation for collaborative practice, (b) the structures of collaborative practice as the domains of collaboration, (c) the key principles of collaboration, and (d) the processes of collaboration. Although we take this framework as the basis in its structures, we have reworked and revised the framework by replacing some of the terms with more appropriate ones and elaborating on the component on the processes of collaboration specifically to make the model focus on the practice involving clinical relationships between persons and professionals as depicted in Fig. 1. The shaded area of the figure refers to the focus of the CDCP Model development.

The first component of this framework is the overall frame orientation for a collaborative practice that consists of person-centered care, recovery-orientation, and a pluralistic orientation. Person-centered care and recovery-orientation are the practice philosophies and perspectives that are key to user-involvement in care processes as these are oriented to achieving mutual understanding, shared decision-making, co-management of care, support of self-management and empowerment, and supportive care. Partnership with persons, dialogue for shared decision-making, and tailoring the care to the person are the key ingredients of these frame orientations [24, 44, 45]. The perspective of person-centered care focuses on the care ‘of the person’, ‘by the person’, ‘for the person’, and ‘with the person’ [46], and the key dimensions underlying person-centered care include (a) holistic, multidimensional perspective for a person, (b) the perspective of client-as-individual, (c) sharing power and responsibility in decision making, and (d) therapeutic alliance [47]. The recovery-orientation, in addition, focuses on the person and his/her everyday life rather than on pathologies or symptoms with the personal unique process as its central core, including person-centeredness, empowerment, purpose, and hope as the key components [8, 48–50]. Recovery-orientation in MHSA care focuses on individuals to attain and maintain active, meaningful personal social lives in the context of mental health problems. In developing the model of care process for MHSA practice proposed in this paper, the perspective of pluralistic orientation [51, 52] has been added to the frame orientation of person-centered practice and recovery-orientation. This has been added because the model focuses on clinical practice, which needs to embrace not only the differences in individualities of persons and professionals but also in terms of options, choices, variations in the modes, methods, and approaches of care and therapy. Pluralistic orientation means that one seeks to have access to as many options on how to collaborate in both selecting specific modes of treatment and care, as well as in applying chosen modes of care and treatment with persons [50]. This orientation points to the possibility and availability of choices in a repertoire of therapeutic approaches and modes of care, in the routes in shared decision-making, and the configurations of MHSA service provision. A pluralistic orientation points out that clinical practice processes must also embrace tailored forms of collaboration through the acceptance and integration of the notion that people are different and contexts of people’s lives and their effects on our choices are varied.

The second component of the framework identifies the domains of collaborative practice specified at three levels: (a) collaboration between persons (and/or families) with professionals and (b) collaboration among service providers (i.e., professionals), and (c) collaboration among service sectors. This specification of the domains emphasizes the critical features in collaborative practice in healthcare that has to encompass both at the individual clinical practice level and the healthcare service provision level involving various service providers and organizational sectors. Our focus of the proposed CDCP Model is oriented in the structure of the collaboration between the person/family and the professional.

The third component of the framework identifies the most essential principles that promote collaboration among people. The key values for collaboration emphasized in the literature are (a) shared value in collaboration, (b) equalization of power in relationships, (c) mutual trust and respect, (d) sharing of visions and goals, (e) developing and maintaining interpersonal alliance, (f) self and mutual understanding, and (g) open communication. Three essential principles encompassing all of these values are included in this framework identified as self-understanding, mutual understanding, and shared decision-making. The principles of self-understanding and mutual understanding form the first-line posture that can commit people for collaboration. Self-understanding encompasses knowing one’s strengths and weaknesses, one’s history, one’s attitudes toward people including self and others, and one’s wishes, hopes and despairs. Such self-understanding is the base from which the directions, courses, and trajectories of clinical processes begin. Self-understanding is critical for all participants in collaborative work as it is the base upon which mutual understanding can develop. On the other hand, mutual understanding is the key principle for collaboration because collaboration begins with the appreciation of and understanding of the other’s needs, goals, and postures in social relationships. Since the discourse in clinical practice is a form of social relationship, mutual understanding of participants (that is, the person and the professional) in interactions of clinical practice is the precondition for collaboration. In clinical practice, interactions involving back-and-forth movements, specified as *turn*-*taking* [25, 53], brings about collaboration emerging through self-understanding and mutual understanding. The third principle in this framework is shared decision-making. All forms of collaborative endeavors are rooted in shared decision-making which is based on the respect and appreciation of each other’s views, postures, and needs concerning what to do, how it is to be done, and where this should lead the participants. In developing the CDCP Model for collaborative practice focusing on the person/professional relationships, we have revised this set of principles specified in the original framework, replacing them with the principles of *mutual understanding, negotiation, and working together*. In our explication of the processes of collaboration in our empirical works reported in Part One of this series, we delineated these three principles as the guiding posts for the application of collaborative processes in practice. Mutual understanding encompasses self-understanding as the pre-condition, while negotiation embeds the principle of shared decision-making but goes further by integrating the concept of consensus building. The principle of negotiation encompasses the openness to other’s perspectives and positions, and the willingness to come to agreements regarding whatever disputes exist in situations. In addition, we found the concept of working together as the major driving force for collaboration in practice. Working together means being partners in accomplishing the work of recovery and getting or remaining well in the context of MHSA care. Working together is based on mutual understanding regarding what needs to be accomplished and how participants will contribute to accomplishing the work together. It is based on an ancient proverb that states: “Even a piece of paper is lighter when lifted by two people together.” Collaborative processes explicated in our work are the ways to bring about the commitments to these three guiding principles in clinical practice.

**Fig. 2:**
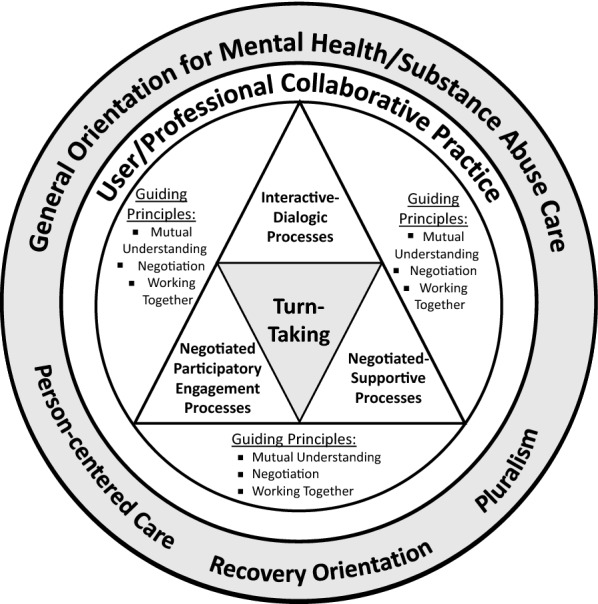
Configuration for the collaborative, dialogue-based clinical practice model (The CDCP Model) for community mental health and substance abuse care

The fourth component of the framework is the specification of the essential processes of collaboration identified as open dialogue and participatory engagement in the original framework. We have reformulated this component to encompass three essential process-types including (a) *interactive*-*dialogic processes* replacing the concept of open dialogue, (b) *negotiated*-*participatory engagement processes* clarifying the concept of participatory engagement, and (c) *negotiated*-*supportive processes* as an additional type of processes through our empirical work presented in Part One of this series. The proposed CDCP Model for community MHSA care focuses on this fourth component through the elaboration of the types of collaborative processes pointing to specific collaborative strategies in each type applicable in practice. These three types of collaborative processes (i.e., interactive-dialogic processes, negotiated-participatory engagement processes, and negotiated-supportive processes) are essential for achieving mutual understanding, negotiation, and working together in person/professional relationships and in bringing about best person outcomes possible in terms of clinical management, recovery, and socially meaningful and active life in the perspective of citizenship. Replacing “open dialogue” with the term, interactive-dialogic process-type is a way to go beyond the limited meaning of open dialogue that has its beginning in the therapeutic application for psychosis and discursive acts [54]. By reformulating this type of collaborative processes designated as “interactive-dialogic processes” we expand the process of interaction between the person and the professional to include various discursive as well as non-verbal interactive strategies beyond those identified in the open dialogue framework. The interactive-dialogic process-type focuses on interactions which are comprised quite heavily of conversations between participants as “unconstrained back-and-forth exchanges of meanings, voices, and interpretations, and of creating shared meaning through which common understandings regarding situations, problems, goals, and approaches are developed and shared” ([20], p. 12). The process-type of negotiated-participatory engagement refers to *active sharing and negotiated involvement* of participants in the work of shared decision making, goal setting, planning, and implementing therapeutic plans and approaches. The processes of negotiated-participatory engagement in clinical practice between persons and professionals also encompass the active involvement of both participants in carrying out activities and interventions by sharing information and resources. The processes of participatory engagement are always negotiated regarding participants’ (i.e., the person and the professional) needs, wants, and goals as well as in terms of their respective strengths, resources, preferences, and responsibilities. The major focus of the processes of negotiated participatory engagement is “working together.” The third additional process-type specified as “negotiated-supportive processes” refers to the ways of supporting persons to attain and maintain active and meaningful social lives, which are the critical aspects of the recovery-oriented MHSA care. From the perspective of collaboration, support has to be based on negotiation and alliance with persons’ perspectives of their needs and goals. The support process focuses on persons’ needs associated with their lives in the contexts of everyday life, in communities and society at large as well as in relation to their being the recipients of healthcare services. Supportive processes as a form of collaboration are rooted in the professionals’ understanding and appreciation of the person’s needs, goals, and wants in everyday lives as well as the person’s difficulties in dealing with the healthcare system, the community, and the society at large. The supportive processes are necessary and critical in the clinical practice of MHSA care addressing persons’ needs and problems that are not directly associated with their mental health problems but are experienced because they are “users” of services and are living in specific personal and social contexts with MHSA problems. In this sense, the supportive processes are not strictly “therapeutic” but are oriented to enhancing users’ quality of life and recovery as persons and social agents. Supportive processes in the context of collaborative practice require the involvement of persons and professionals in a concerted effort to bring about socially active and meaningful lives for the persons. Table 1 lists specific collaborative strategies in the three types of processes that are integrated into the proposed CDCP Model for community MHSA care reported in Part One of this series. The proposed CDCP Model is specifically at the level of person/professional collaboration and is depicted in Fig. 2 anchored in the framework by Ness et al. [20] incorporating the revisions and additions discussed above.

**Table 1:** Collaborative strategies in three types of processes

Process types for collaboration	Specific collaborative strategies
Interactive-dialogic process	Maintaining human relationship Walking alongside Information sharing Seizing the present moment Taking the perspective of the other Aligning & Scaffolding
Negotiated-participatory engagement process	Feedback-informing process Putting differences to work Negotiated partnering Accommodating user participation Addressing the tension between help and control
Negotiated supportive process	Helping in context Coordinating Pulling together Advocating Availing

The CDCP Model for community MHSA care depicted in Fig. 2 incorporating the collaborative strategies listed in Table 1 for three types of collaborative processes is applicable in clinical practice involving encounters with users and/or users’ family members (or significant others) involved in the care of users of MHSA services. Clinical encounters between users (and family members) in community MHSA care can be characterized in many ways according to the types of users’ needs for services such as prevention, MHSA episodes, continuing care/service, or crises, and may occur at various healthcare settings as well as at users’ homes. While specific dynamics of various clinical encounters may vary in their contents and progression, the basic assumption is that collaboration between users and professionals in clinical encounters is one of the key processes that are critical in bringing about the best outcomes in users. The proposal for the application of the CDCP Model in MHSA care requires professionals to become knowledgeable about the framework and competent in the application of the collaborative strategies of these three process-types in their clinical encounters with users and/or family members.

In the following section, we present a clinical case illustrating the application of the CDCP Model for community MHSA Care. This illustration is a description of the case, in which one of the authors was a member of the therapeutic team, reflecting upon the CDCP model.

## Clinical Application of the CDCP Model for Community MHSA Care—an illustration

The case illustrated is reconstructed from the clinical notes of an adolescent user at a municipal unit for mental health for children and adolescents. A pseudonym is used and no personal information is included in this scenario in order to protect the identity of the user. Joe has dropped out of school and he has been referred to the family therapy team, a team that is part of Mental Health for Children and Adolescents in a Norwegian city. Joe is 14 years old. The practices of this team can be seen as exemplifying the CDCP-model. This team is *person*-*centered* in the sense that in any meeting and treatment session each person`s perspectives and concerns must be voiced and heard. This requires each person to be seen and understood in their context of living and predicaments of life. Central in the person-centered perspective of this team is to acknowledge that personhood of the single individual arises only as person-in-context. Any attempt to see the person as a member of a group based on a descriptive, diagnostic, theoretical or other categorization, means a reduction of the unique personhood of each individual. In the case of Joe, non-diagnostic and non-theoretical descriptions were, therefore, the continuous ways of focusing on him as a person, at the same time assuring his rights for diagnosis and assessments. This was done by inviting Joe and especially his family to tell their personal stories around Joe falling out of school and any other parts of their life stories that they felt were important. Together with this orientation, *the recovery orientation* sought to strengthen this through a focus both on the rights of Joe and his family, and on securing service-user participation on all levels of the contact between Joe, the family, the team, and other collaborative partners. The *recovery orientation* was applied for capacity building to promote human rights and to combat stigma and discrimination. Furthermore, the *recovery orientation* was the base for the application of various strategies to strengthen Joe, his family, and his social network for their participation in choice and decision-making. The *recovery orientation* also laid out the foundation for supporting the development of civil society movement to conduct advocacy and influence policymaking at various institutional levels critical for Joe’s recovery [55]. Securing participation on all levels of contact between Joe, the family, the team, and other collaborative partners were emphasized. Throughout the contact with Joe and his family, *the pluralistic orientation* was exemplified by the therapists’ use of different tools, techniques, manners of thinking and being together, which were evaluated together with Joe and his family as helpful. We used Routine Outcome Monitoring [56] as the central collaborative activity with Joe and his family to obtain their feedback on the processes and outcomes and also to secure their rights and participation in the care processes. Falling out of school is a type of event that mobilized great fear and concern both in parents, therapists, school personnel, social services, and other concerned citizens. This points to the second component of the CDCP-model; *user/professional collaborative practice*. In the first part, the collaboration between Joe and the family and the therapists of the team, developed in a struggling manner because Joe was very reluctant to talk with any of the therapists in the team. Due to prior experiences with mental health services he simply stated that: “I have had enough of such people. They only make things worse.” Based on this message, it was decided that the therapists talked with Joe`s parents and did not intrude on him. The parents had two main concerns outside the situation of not attending school. The first was collaborative problems with personnel from the school where they both felt accused of mishandling their son. They had a contrary experience in which they believed the mishandling was happening in the school. They stated that something had to be done with both their and Joe`s relationship to the school. The other concern was that they were worried that something had happened to Joe outside of the school, bullying or some other incident that had scared Joe. They described their son as a bit timid and socially awkward and felt that he needed help with both such a possible incident and his sociality in the world. The parents and the team’s therapists jointly decided for the team to contact the school to arrange for a meeting. The parents wanted therapists to have this meeting without them for the therapists to make up their own opinions. This exemplifies the second part of this component of the model. A meeting was held between the principal, teachers, school social- and health care personnel. The beginning of the collaborative process was established and the care process for Joe was established in the mode of collaboration. The third component of the CDCP Model points out the most essential principles that promote collaboration among people: *mutual understanding, negotiation, and working together*. For the therapists, it became important to make more visible for all involved participants the diverse understandings and positions in the situation of Joe, his family, and the school. A simple principle of practice was guiding the therapists: all perspectives are relevant and speak to import parts of the predicament of all involved persons. This invites conversation around how each understands one’s own and the situation of all the other participants. Further that any decision that touches upon any of the participants must be a shared decision involving those touched by the decision. To reach such a goal, negotiations become the central part of conversations with the aim to be able to work together toward common goals.

The realization of these three principles happens through the actual and practical work within the fourth component of the CDCP Model: *the collaborative process types and their specific collaborative strategies*. In the situation of Joe, his family and the school, the following can be marked as especially important: Any meeting is built on the ability of the therapists to create an opportunity for *turn*-*taking*. What became clear was that it would be important for everybody if somebody could involve Joe in such turn-taking conversations as an *interactive*-*dialogic process.* It happened that Joe was at the beginning of realizing his interest in learning to play the guitar. This opened up the opportunity for the music teacher to come to Joe’s home and help him with his learning objective concerning the guitar. In this situation Joe told about bullying incidents and that he felt anxious about going outside the house. The teacher asked if he could invite in one of the therapists of the team to talk about this. This therapist also played the guitar and through collaboration around playing the guitar, this opened up possibilities for Joe, the teacher, and the therapist to talk also about the effects of the bullying and what to do about it. As a consequence of this discovery, conversations on bullying spread to all meetings involved in the work with Joe and from there into the whole school milieu. The *specific collaborative strategies* important in this situation were *walking alongside*, *seizing the present moment,* and *aligning and scaffolding.* Especially the use of the feedback tool involving feedback informing process with Joe as an *informed*, *service*-*user involved process* contributed to the collaborative work exemplifying *the negotiated*-*participatory engagement process* of the CDCP Model. *The interactive*-*dialogical process* established in various meetings sought to maintain all the relationships as a human relationship of respect and equal power. Respect and equality were sought and maintained by the therapist keeping everybody informed of what was being done and explicitly stating their understanding of all the other participants’ perspectives. When discrepancies between these perspectives and understandings were discovered, these were rectified and corrected through discussions. Again, the use of formalized feedback exemplified the presence of *the negotiated*-*participator engagement process* with a clear weight of putting all the different perspectives to work and that accommodating user participation meant that everybody should have a say in decisions that touched upon their situation. Lastly, the strategies in *the negotiated supportive process* were applied in this situation as well. The strategy of “helping in context” was applied in having the first meeting of the therapist with the school personnel without the presence of the parents following the request of the parents and also in organizing the help needed by Joe for guitar lessons. Coordinating was evident in the team’s concerted work involving the parents, the therapists, and the school personnel to provide multi-faceted approaches to help Joe, while “pulling together” was apparent in the efforts to help Joe’s problems with bullying involving all members. The therapists along with the music teacher advocated for Joe specifically to the school to deal with not only the problems of bullying of Joe but bullying in school in general. “Availing” was apparent in the willingness for the music teacher to give lessons to Joe, and the therapeutic team’s approach in working with Joe and his family.

In order to provide help concerning the family’s economic problems, the therapists took part in helping to get economic support from social services. The school needed to attend to the bullying situation which was discovered not only to have affected Joe but other adolescents too. The therapists took part in meetings on anti-bullying work and supported through the specialist’s declaration that for a period some boys, who perpetrated the bullying, needed extra teaching resources from the psychological-pedagogical services as support of the teachers in their anti-bullying work.

The contact with Joe, his family, and his school continued for almost 2 years. After about 8 months into the collaboration, Joe started to attend school again. In the following period, he got some therapeutic help with his anxiety and social awkwardness. The bullying was now a collective concern of the whole school and all the students and teachers were involved in the anti-bullying work so Joe was no longer a special case, but one of many who had felt the effects of bullying, something of which he expressed appreciation. The worries of the parents were lowered and their situation around the economy was resolved as well. Six months before ending contact there was only one meeting to decide if there were reasons to continue the contact. Both parents and school wanted to have the possibility of contacting the team if there was any change back to the original situation. During the ensuing 6 months there was no contact and through telephone calls to all involved participants, it was decided that the contact between Joe, the family, the school and the team would be terminated, closing the case.

## Discussion and Closing remarks

The CDCP Model presented in this paper has been developed through the analytic and empirical findings in the literature and the authors’ work in the development of MHSA practice in the community. It fills the gap that exists in the field of community MHSA practice regarding how to operationalize systematically the tenets of person-centered, recovery-oriented, and pluralism-oriented practice in terms of user/professional collaboration. The CDCP Model specifies the guiding principles of mutual understanding, negotiation, and working together as its operational foundation, and identifies three types of collaborative processes as interactive-dialogic processes, negotiated participatory engagement processes, and negotiated supportive processes. For these sets of processes, specific types of collaborative strategies have been identified by integrating and consolidating the empirical findings of the authors’ work, which were presented in Part One of this series. This is a comprehensive model of collaborative practice applicable in MHSA practice, especially in the context of community-based care.

The CDCP Model thus can be applied in practice in two specific ways: (a) prospectively as the basis for designing and carrying out clinical services to people, and (b) retrospectively as a reflective analytic tool to evaluate one’s practice or to assess the quality of MHSA practice in terms of collaboration. The prospective use of the CDCP Model in practice would involve an introduction to the CDCP Model and training for the application of the Model in specific clinical situations. This will involve training for the integration of the foundational bases of the CDCP Model and its guiding principles into professionals’ belief systems and for the development of competency in the application of various strategies identified as three types of collaborative processes in the Model. Integration of the CDCP Model into various recovery-oriented and person-centered practice designs for MHSA care will enrich their designs with the explication of the CDCP Model in terms of its foundations, guiding principles, and collaborative strategies. The retrospective use of the CDCP Model can be in the form of reflective practice [57] in which professionals evaluate their practice by examining their practice vis-à-vis the components of the CDCP Model. The CDCP Model can be used as the template for developing an evaluation tool for collaborative practice in MHSA care as well. The Model also can be applied in assessing professionals’ practice in research that explores and/or examines the effectiveness of user/professional collaboration in MHSA practice.

There are, however, limitations regarding the generalizability of the CDCP Model as this model has been developed incorporating the results of a meta-synthesis carried out with a set of studies by one group of researchers with the same perspective. The general applicability of the CDCP Model in various practice settings may be limited due to the diversity in the sorts of community mental healthcare settings present in different societies and the dynamics of user-professional relationships shaped by sociocultural factors such as multiculturality, an existence of disfranchised population, or the culture of dominance in healthcare relationships. However, as Noblit [58] in citing Van der veer [59] states that the conceptual models we create are not universally applicable but are universalized to have an impact, the CDCP Model can be viewed as the “universalized” beginning to have an impact on the practice of community mental health care.

To address this limitation and also to extend the knowledge further, the CDCP Model needs to be refined and elaborated further, especially by identifying other collaborative strategies not specified in the CDCP Model, as there would be both general and context-specific strategies that would be useful for user/professional collaboration.

## Data Availability

No empirical data is used in this paper.

## Abbreviations

MHSA: Mental health and substance abuse)
CDCP Model: Collaborative dialogue-based clinical practice model)
OD: Open dialogue)
